# Genetic Dissection of Acute Ethanol Responsive Gene Networks in Prefrontal Cortex: Functional and Mechanistic Implications

**DOI:** 10.1371/journal.pone.0033575

**Published:** 2012-04-12

**Authors:** Aaron R. Wolen, Charles A. Phillips, Michael A. Langston, Alex H. Putman, Paul J. Vorster, Nathan A. Bruce, Timothy P. York, Robert W. Williams, Michael F. Miles

**Affiliations:** 1 Department of Human and Molecular Genetics, Virginia Commonwealth University, Richmond, Virginia, United States of America; 2 Department of Pharmacology and Toxicology, Virginia Commonwealth University, Richmond, Virginia, United States of America; 3 Department of Neurology, Virginia Commonwealth University, Richmond, Virginia, United States of America; 4 Department of Electrical Engineering and Computer Science, University of Tennessee, Knoxville, Tennessee, United States of America; 5 Department of Anatomy and Neurobiology, University of Tennessee Health Sciences, Memphis, Tennessee, United States of America; University of Chicago, United States of America

## Abstract

**Background:**

Individual differences in initial sensitivity to ethanol are strongly related to the heritable risk of alcoholism in humans. To elucidate key molecular networks that modulate ethanol sensitivity we performed the first systems genetics analysis of ethanol-responsive gene expression in brain regions of the mesocorticolimbic reward circuit (prefrontal cortex, nucleus accumbens, and ventral midbrain) across a highly diverse family of 27 isogenic mouse strains (BXD panel) before and after treatment with ethanol.

**Results:**

Acute ethanol altered the expression of ∼2,750 genes in one or more regions and 400 transcripts were jointly modulated in all three. Ethanol-responsive gene networks were extracted with a powerful graph theoretical method that efficiently summarized ethanol's effects. These networks correlated with acute behavioral responses to ethanol and other drugs of abuse. As predicted, networks were heavily populated by genes controlling synaptic transmission and neuroplasticity.

Several of the most densely interconnected network hubs, including *Kcnma1* and *Gsk3β*, are known to influence behavioral or physiological responses to ethanol, validating our overall approach. Other major hub genes like *Grm3, Pten and Nrg3* represent novel targets of ethanol effects. Networks were under strong genetic control by variants that we mapped to a small number of chromosomal loci. Using a novel combination of genetic, bioinformatic and network-based approaches, we identified high priority cis-regulatory candidate genes, including *Scn1b*, *Gria1*, *Sncb* and *Nell2*.

**Conclusions:**

The ethanol-responsive gene networks identified here represent a previously uncharacterized intermediate phenotype between DNA variation and ethanol sensitivity in mice. Networks involved in synaptic transmission were strongly regulated by ethanol and could contribute to behavioral plasticity seen with chronic ethanol. Our novel finding that hub genes and a small number of loci exert major influence over the ethanol response of gene networks could have important implications for future studies regarding the mechanisms and treatment of alcohol use disorders.

## Introduction

Alcohol use disorders (AUD) are extremely prevalent, with an estimated 18 million Americans meeting diagnostic criteria for an AUD (2007 Survey on Alcohol and Drug Use). However, only a small subset of the wider population that regularly consumes alcohol will ever meet clinical criteria for alcohol abuse or alcoholism. AUD susceptibility is strongly influenced by genetic factors, accounting for as much as 40–60% of the risk for developing an AUD [1], [2]. While population and family-based association studies have discovered a number of genetic markers linked to AUD susceptibility [3], [4], [5], the highly complex and multifactorial nature of the disorder suggests that, independently, each of these associations accounts for only a small portion of the overall genetic variance. Moreover, the molecular mechanisms underlying the neuroplasticity accounting for AUD likely involves networks comprised of many more genes than currently identified as affecting behavioral responses to ethanol in animal models or genetically associated with AUD in humans.

Most experimental approaches to studying complex traits such as AUD have focused on identifying the role for “single genes” even when employing genome-wide tools such as microarrays. More recently, microarray expression data has been used in systems genetics studies to construct maps of gene interactions on the basis of correlated expression patterns, providing unprecedented insight into the molecular networks underlying complex traits. Such network-based approaches may prove to be more effective for delineating genetic factors underlying individual variation in AUD risk and the neurobiology of ethanol and drug abuse disorders. A gene network (a group of genes that are coordinately regulated or functionally inter-related) producing significant influence on neural pathways affecting ethanol-associated behaviors is both more likely to be experimentally detected across human and animal studies and may also identify key nodes that could serve as rationale therapeutic targets.

Mining microarray expression data for patterns of correlated gene expression (co-expression), has made it possible to identify novel gene/gene interactions [6] and construct non-parametric models of gene transcription networks. Extending these analyses to include genotypic data allows key regulators of a gene network to be implicated by scanning for associations between DNA variation and co-regulated groups of genes [7]. Such network-based genetical genomics approaches have previously been utilized to characterize the gene expression architectures of yeast [8], mouse liver [9] and the nervous system [10]. Investigators have also used this approach to dissect a variety of mouse models for complex traits, including alcohol preference [11], susceptibility to obesity [12], type 2 diabetes [13] and tumorigenesis [14].

Initial responses to ethanol are highly informative predictors of AUD risk, with level of response (LR) being inversely correlated with susceptibility [15], [16], [17]. Similar inverse relationships between acute LR and ethanol consumption have been observed with a number of selectively bred or gene targeted rodent strains [18]. Therefore, an understanding of the molecular pathways initially perturbed by ethanol may identify important contributors to LR behaviors and elevated AUD susceptibility. As with humans, genetically diverse populations of mice exhibit a wide range of ethanol sensitivity. The B6 and D2 inbred strains are a particularly well documented example, with numerous studies reporting that ethanol induces significantly larger responses in D2 mice, when compared to B6, across a number of measures such as locomotor activation [19] and withdrawal severity [20].

Our prior work showed that acute ethanol administration (2 g/kg, 4 hours) induces regionally-selective changes in gene expression in the mesocorticolimbic system [21]. In all profiled brain regions Kerns et al. [22] found the ethanol induced response of these genes was generally markedly different between B6 and D2 mice. The greatest disparity in transcriptional LR was in the prefrontal cortex (PFC), where far more genes were regulated by ethanol in D2 mice than in B6 mice. However, these prior studies on two strains did not have sufficient power for robust definition of gene correlation networks or genetic analysis of mechanisms underlying the ethanol-responsive gene sets.

In order to extract and dissect acute ethanol-responsive gene networks, we performed a large-scale gene expression analysis across recombinant inbred (RI) strains derived from the B6×D2 (BXD) genetic mapping panel. The BXD family has been widely used for both genetic studies on ethanol behaviors and many other phenotypes, and for expression genetics studies [10]. For each included BXD strain, we profiled PFC, ventral midbrain (VMB) and nucleus accumbens (NAc) transcriptomes of mice from saline and ethanol treatment groups. This produced the most extensive assessment of ethanol-responsive brain gene expression to date. Furthermore, we focused on PFC and produced the first genetic analysis of ethanol-responsive gene networks. Our results show network-level enrichment of genes involved in synaptic plasticity and identify key hub genes regulating the ethanol response for large networks of genes. This first such detailed genetic analysis of the acute “ethanol responsome” may provide valuable insight for molecular mechanisms underlying the neurobiology of ethanol and also ultimately provide novel AUD susceptibility candidate genes and targets for intervention in alcoholism.

## Methods

### Ethics Statement

All animal procedures were approved by Virginia Commonwealth University Institutional Animal Care and Use Committee under protocol numbers AM10332 and AM10139, and followed the NIH Guide for the Care and Use of Laboratory Animals (NIH Publications No. 80–23, 1996).

### Animals and tissue collection

All BXD RI strains and the B6 and D2 progenitors were purchased from Jackson Laboratory (Bar Harbor, ME). All animals were male and between 10–12 weeks of age. Mice were housed 4 per cage with *ad libitum* access to standard rodent chow (catalog #7912, Harlan Teklad, Madison, WI) and water. Following a two week acclimation period mice were injected intraperitoneally (IP) with saline or 1.8 g/kg of ethanol. This ethanol dose was originally chosen from pilot experiment data to maximize anxiolytic activity and minimize sedative responses (decreased locomotor activity) as part of a parallel study of ethanol induced anxiolysis. In that study, all mice underwent behavioral testing that included 15 minutes of restraint in a 50 mL conical tube followed by 10 minutes in a light-dark chamber. The results of these behavioral genetics experiments will be published elsewhere (Putman et al, submitted) and are not discussed in this manuscript. Mice were killed by cervical dislocation four hours following IP injection. Immediately thereafter, brains were extracted and chilled for one minute in iced phosphate buffer before being microdissected into 8 constituent regions as described previously [21], including medial prefrontal cortex, nucleus accumbens and ventral midbrain, which includes ventral tegmental area and substantia nigra. Excised regions were placed in individual tubes, flash-frozen in liquid nitrogen and stored at −80°C.

### Microarray data generation

This study incorporated prefrontal cortex tissue from 27 BXD strains, nucleus accumbens and ventral midbrain tissue from 35 BXD strains, as well as B6 and D2 tissue from all three regions. Frozen tissue for a given brain region and strain was pooled from 4–5 animals and homogenized with Aurum™ total RNA fatty and fibrous tissue extraction kit (BioRad, catalog #732–6830) and a Tekmar homogenizer. RNA concentration was determined by absorbance at 260 nm, and RNA quality was analyzed by electrophoresis on a Experion analyzer (BioRad, Hercules, CA) and 260/280 absorbance ratios. All RNA samples had RNA quality indices (RQI)≥8. Total RNA (5 µg) derived from each pool and spike-in poly-A RNA controls were reverse transcribed into double-stranded cDNA using Affymetrix SuperScript® one-cycle cDNA kit (Invitrogen, catalog #A10752030). Biotin-labeled cRNA was synthesized from cDNA using the GeneChip IVT labeling kit (Affymetrix, part #900449) according to manufacturer's instructions, purified using the RNAeasy Mini Kit (Qiagen, Mountain View, CA), and quantified by absorbance at 260 nm. Labeled cRNA samples were hybridized to Mouse Genome 430 2.0 microarrays (Affymetrix, part #900497) according to the manufacturer's protocol and as described previously [21]. The number of microarrays involved in this study required that their processing be divided in batches of manageable sizes. To avoid systematic variation of expression data through technical batch effects, we performed a supervised randomization of samples into batch groups prior to each of the following processing stages: total RNA extraction, cRNA synthesis and hybridization. Both a saline and ethanol-treated mouse from a single strain were always processed together to minimize risk of technical variation confounding ethanol response detection. Annotation data for Mouse Genome 430 2.0 probe-sets was obtained from the GeneNetwork Data Sharing Zone (genenetwork.org/share/annotations).

### Microarray analysis

Microarray quality was assessed by inspecting the distributions of log-transformed probe intensity values, as well as scanning for outlier chips using a standard battery of quality measurements, including: average background, scaling factor, percentage of probe-sets called present and 3′/5′ ratios for *Actin* and *Gapdh*. Bioconductor's implementation of the MAS 5.0 Detection Calls Algorithm, available in the affy package [22] for R [23], was used to generate absent/marginal/present calls across all samples. We excluded any probe-sets called absent in ≥95% of samples from all subsequent analyses to improve the ratio of true positives in downstream statistical filtering [24]. This removed 14,096, 12,970 and 13,312 probe-sets from the PFC, NAc and VMB, respectively. The lists of ‘absent’ probe-sets were largely overlapping, with 11,343 probe-sets filtered out of all 3 regional datasets, suggesting this filtering step largely removes probe-sets targeting genes unexpressed in brain tissue. Expression data from the saline and ethanol treatment groups were background corrected, quantile normalized and summarized using the robust multi-array average (RMA) expression measure [25]. For analysis of SNPs possibly affecting microarray probe performance, the D2 genome sequence provided by Xusheng Wang in Dr. William's laboratory was used to identify probes overlapping a B6/D2 SNP (Table S8). Affymetrix probe sequences were aligned to the mm9 version of the mouse genome provided by Bioconductor, using the Biostrings package for R. All datasets generated for this paper can be queried on GeneNetwork (genenetwork.org) or downloaded in their entirety from the Gene Expression Omnibus repository under accession number GSE28515. All data is MIAME compliant.

### Identification of ethanol-responsive genes

The large scale of this study made cost prohibitive the inclusion of biological replicates for each RI strain across treatment groups. Therefore, assessing the reproducibility of changes in gene expression within a single strain by conventional methods, such as SAM [26], was not possible. We therefore used an alternative approach to identify probe-sets with extreme ethanol expression changes across a minority of strains or smaller but consistent changes across a larger portion of the BXD family. The impact of acute ethanol on transcript abundance was measured using the Significance-score (S-score) algorithm [27], which utilizes individual probe-level data to determine the statistical significance of transcript level differences between a pair of Affymetrix microarrays. We utilized the R implementation of the S-score algorithm [28] to compare microarray expression levels within BXD strains across treatment groups to generate a saline vs ethanol S-score for each probe-set, where a positive S-score indicates up-regulation with ethanol and vice-versa. In the case of the progenitor strains, where biological replicate microarrays were available for each strain in triplicate, S-scores were generated using the SScore function's class label feature.

S-scores are normally distributed with a mean of 0 and a standard deviation of 1 [27]. For 2-tailed tests, p-values for each probe-set were calculated as twice the probability of obtaining an S-score at least as large as the absolute value of the observed S-score. Statistical significance of a given probe-set's ethanol response across BXD strains was assessed using Fisher's combined probability test [29]. An R implementation of Fisher's method, available as part of the MADAM package [30], was used to combine the S-score transformed p-values. This process was then repeated for 1,000 random permutations of the observed S-score expression matrix, so that empirical p-values could be obtained by comparing observed results to the permutation distribution. Finally, to correct for multiple testing, q-values were generated from the empirical p-values [31]. Probe-sets with q-values≤0.05 were considered to be significantly ethanol-responsive.

### Paraclique formation and network analysis

Steady-state RMA and saline vs ethanol S-score expression datasets were analyzed using a graph theoretical algorithm [32] to identify gene co-expression networks. We first calculated all pairwise Pearson correlations across probe-sets, where each probe-set is represented as a vector of BXD expression values, and used this data to construct an unweighted graph in which vertices represent probe-sets and edges were present whenever the absolute value of the correlation between two probe-sets was ≥0.7. The choice of threshold when converting a weighted graph to an unweighted graph is analogous to the choice of p-value when determining significance; it is chosen to produce a reasonable tradeoff between false positives and false negatives. A correlation threshold of |0.7| across 27 strains yields a correlation p-value of 4.8e-05 (calculated using Student's t-distribution). Such low p-values are indicative of the rigor of graph theoretical techniques.

The most natural grouping of vertices in a graph is by cliques, or fully connected subgraphs. While finding the maximum clique is a well-known computationally intractable problem, being *NP-complete*, the topology of biological graphs lends itself to solution by advanced algorithmic implementations [33], [34]. Since the inevitable noise in large microarray datasets can render clique too restrictive, we used a relaxed version termed a “paraclique”. For graphs constructed using a correlation threshold, we iteratively extracted maximum cliques and used them as cores to build paracliques. A paraclique starts with a maximum clique and gloms onto all vertices with at least some proportion of edges to that clique. This proportion is called the “proportional glom factor.” As a paraclique was formed, the number of edges that must be present for a vertex to be included was scaled to the size of the starting clique. We selected a glom factor of 0.7 for the analyses presented here, which maintains an edge density >90% in nearly all the resulting paracliques. For such defined paracliques, probe-sets had expression responses to ethanol correlated with at least 70% of the other paraclique members at a threshold ≥|0.7|. Lowering the glom factor below 0.7 resulted in a sharp drop-off in edge density. Furthermore, empiric testing showed that more stringent glom factors produced similar overall functional results but tended to fragment known correlated gene groups (e.g. dopamine signaling genes) into multiple paracliques (data not shown).

The relative importance of each node within a paraclique was assessed using network topological measures of connectivity and centrality. Degree of connectivity was equal to number of edges linking a probe-set to other paraclique members, based on the |0.7| edge correlation threshold used to construct the unweighted graphs. Betweenness centrality measures how frequently a node is included in the shortest paths between all pair-wise members of a network. With the edge threshold at |0.7|, Spearman's rank correlations were typically >0.9 between centrality and connectivity. Increasing the edge correlational threshold to |0.9| reduced the connectivity/centrality correspondence to ∼0.6 and greatly increased the centrality for a subset of nodes situated between densely inter-connected subnetworks. We therefore used betweenness centrality scores within unweighted graphs constructed using the more stringent |0.9| edge threshold as a supplemental measure of node importance. Both measures were calculated using the igraph package for R [35].

Fisher's exact test was used to identify paracliques that harbored a greater number of significantly ethanol-responsive probe-sets than what would be expected by chance. The 30,941 probe-sets that passed the present-call filter served as the background for this analysis. Paracliques with a Bonferroni adjusted p-value≤0.05 were judged to be significantly enriched for ethanol-responsive probe-sets.

### Functional analysis

Functional enrichment analyses were performed using ToppFun, a functional enrichment application available at toppgene.cchmc.org as part of the ToppGene suite of web applications [36]. Each paraclique was considered on an individual basis. Entrez ID's for all members of a paraclique were submitted and analyzed for over-representation of genes that belong to a Gene Ontology (GO) category (cellular component, molecular function and biological process), biological pathway, gene family or, similarly, encode a particular protein domain. In order to enhance the specificity and informativeness of these results, we considered only those categories that comprise greater than 3 and fewer than 300 genes, inclusive. Multiple testing was accounted for using a 1% FDR threshold. Results were curated by excluding categories with gene lists more than 80% redundant with other, less enriched, categories.

### Phenotype correlations

We used GeneNetwork's database of phenotypes to identify associations between paracliques and physiological or behavioral traits previously assayed with the BXD population. This analysis was conducted by calculating correlations between GeneNetwork phenotypes and ‘synthetic traits’ used to represent the expression variation of paraclique trans-bands. These synthetic traits were generated by principal component analysis of centered and scaled probe-set expression values. The principal component (PC) trait accounting for the largest proportion of expression variance was used as a single synthetic PC-trait representing the corresponding paraclique trans-band. For each PC-trait, sample order was permuted 1,000 times and correlated with the BXD phenotype database. The permuted correlation distributions were then used to adjust each observed phenotype/PC-trait correlation's p-value.

### Genetical genomics analysis

Quantitative trait locus (QTL) mapping was performed for the saline and ethanol treated RMA datasets, as well as the saline vs ethanol S-score dataset, using a subset of informative microsatellite and SNP markers that have been used to genotype the BXD family [37], [38], and are available from GeneNetwork (genenetwork.org/genotypes/BXD.geno). Linkage between genotypes and expression phenotypes was assessed by performing Haley-Knott regression using R/qtl [39]. Genome-wide adjusted p-values were derived using distributions of maximum LOD scores obtained from 1,000 permutations of each probe-set's expression data. We classified the significance of an expression QTL (eQTL) using guidelines put forth by the Complex Trait Consortium for mapping traditional QTL [40]; where ‘significant’ refers to genome-wide corrected p-values≤0.01 and ‘suggestive’ refers to p-values≤0.63. Estimates of true QTL location were obtained using R/qtl's to calculate 1.5 LOD score drops, as recommended by Manichaikul [41]. Expression QTL were considered *cis* eQTL if their peak chromosomal location was less than 5 Mb upstream or downstream of the regulated gene; all others were considered *trans* eQTL.

Trans eQTL enriched loci, referred to as trans-bands, were detected by splitting the genome into 10 Mb bins and counting the number of suggestive eQTL that mapped to each. In order to determine whether a particular genome bin harbored more eQTL than would be expected by chance, we performed 10,000 permutations, each involving random assignment of all eQTL to a genetic marker and recording the number of mappings at the most populous bin. Observed trans-bands were deemed significant if they exceeded the 95th percentile of the distribution of peak trans-bands captured from each permutation. To facilitate the search for candidate regulators underlying these eQTL enriched regions, we defined support intervals for each of the major trans-bands by aggregating the support intervals calculated for the individual eQTL comprising each trans-band. Trans-band support intervals were defined as the chromosomal regions flanked by genetic markers that were included in at least 80% of the trans-band member's individual support intervals.

### Prioritizing positional candidate genes

Candidate regulators for trans-bands were derived by an empiric ranking scheme for genes contained within the support interval of the trans-band. As detailed in Figure S1, this ranking scheme assigned points for gene information within four categories: genetic sequence variation (SNPs), expression genetics (*cis* eQTL), ethanol regulation and network properties. Positional candidates were scored based on harboring polymorphisms between the B6 and D2 genomes that may alter protein function, which we identified using GeneNetwork's SNP browser. Genes carrying non-synonymous or functional polymorphisms were considered higher priority candidates. We also took into account non-coding polymorphisms whose functional impact may only manifest at the transcript level by further prioritizing interval candidate genes associated with a robust cis eQTL (see above) in either the basal saline or S-score expression datasets. In order to prevent false positive *cis* eQTLs from being prioritized, probe-sets with *cis* eQTL were penalized if their binding target region contained a B6/D2 polymorphism. As Affymetrix probe sequences were designed against the B6 genome, probe SNPs primarily reduce binding avidity with D2 transcripts. Therefore, this penalty was only applied to *cis* eQTL if B6 was the increaser allele. A full list of SNPs identified within probe sequences are contained in Table S8. Candidates were prioritized further if they belonged to the same network as constituents of the linked trans-band, taking into account the relative importance of a gene in the resident network by using the connectivity and centrality measures from the hub gene analysis. Genes identified as significantly ethanol-responsive across the BXD lines received additional scoring.

### Data visualization

Network figures were rendered using Cytoscape [42]. All other figures were generated in R [23] using ggplot2 [43].

## Results

### Identifying ethanol-responsive genes

Previously, we reported an initial microarray analysis of prefrontal cortex (PFC), nucleus accumbens (NAc) and ventral midbrain (VMB) brain regions from the B6 and D2 inbred strains and identified 307 genes that changed significantly with acute ethanol treatment [21]. To extend those prior efforts and construct gene expression network correlations with ethanol behaviors, we conducted an extensive microarray analysis of PFC, NAc, and VMB across 27 recombinant inbred mouse lines from the BXD family and the B6 and D2 parental lines from which they were derived. The greater statistical power and genetic diversity provided by the BXD microarray data made it possible to detect lower magnitude or more variable changes in expression, as well as changes that would otherwise be absent in a study limited to the B6 and D2 strains due to epistatic suppression.

**Figure 1:**
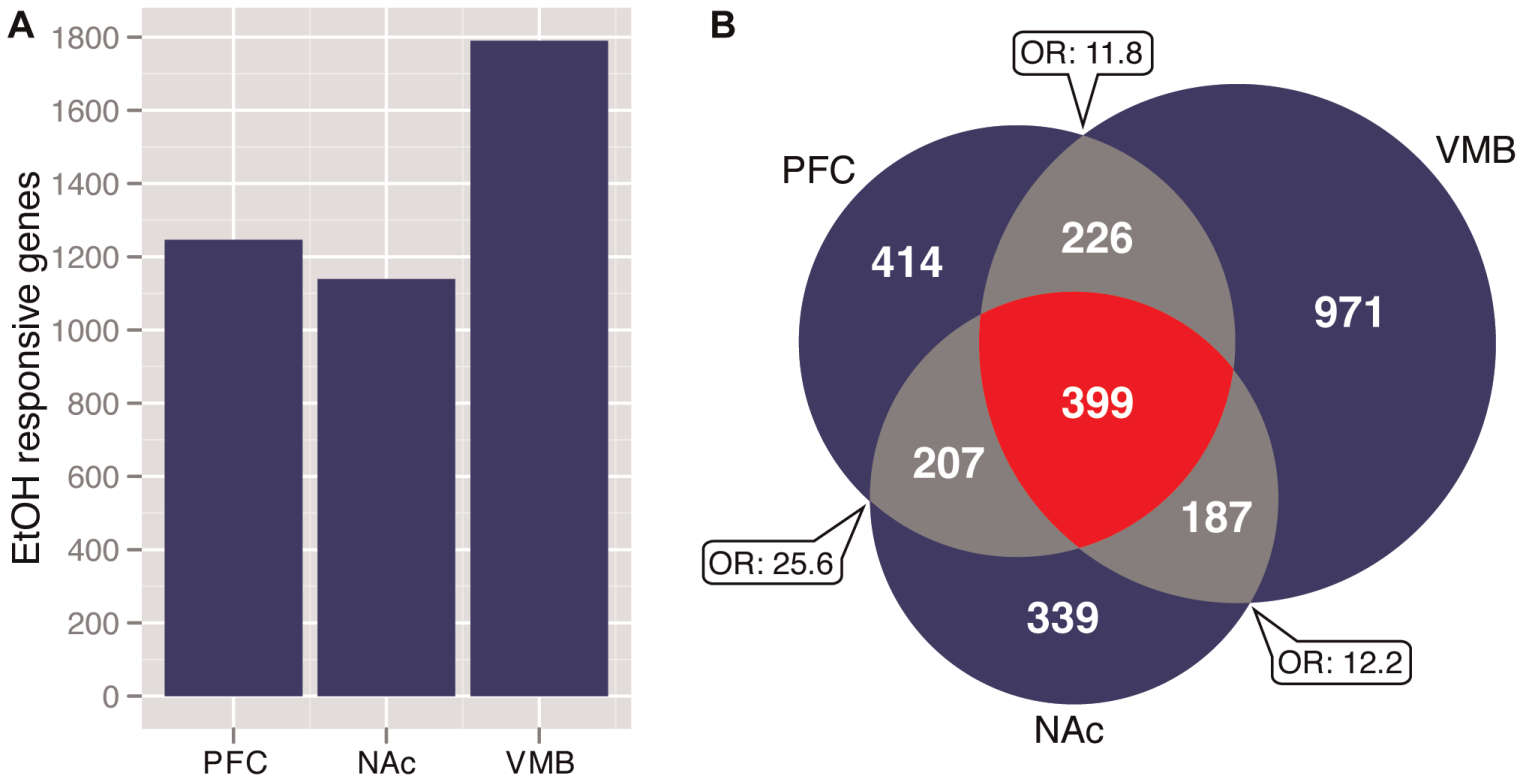
Transcriptional response to acute-ethanol within 3 regions of mesocorticolimbic reward circuit across the BXD family. (**A**) Number of genes found to be significantly ethanol-responsive in the prefrontal cortex (PFC, n = 29), nucleus accumbens (NAc, n = 37) and ventral midbrain (VMB, n = 37) by analysis of saline vs ethanol S-scores across BXD, B6 and D2 samples. (**B**) Venn-diagram depicting which subsets of ethanol-responsive genes are region specific (blue), overlap across two regions (grey) or common to all three regions (red). All three pairwise overlap combinations were statistically significant as determined by Fisher's Exact Test for count data. Odds ratios from this analysis are depicted in word bubbles.

As described in the Methods, we used the S-score algorithm for probe-level analysis of each strain's transcriptional response to ethanol, followed by Fisher's combined probability test. This approach favors genes that consistently responded to ethanol across numerous BXD strains, regardless of direction, rather than genes that exhibited large differences in only a small subset of strains. Analysis of microarray datasets for PFC, NAc and VMB identified 3,512 probe-sets, corresponding to 2,743 unique genes, that changed significantly with ethanol in at least one brain region (Table S1). While not meant as a direct comparison due to differences in strains or directionality, these gene lists contained over 40% of the genes previously identified as ethanol-responsive by Kerns et al. (2005), despite differences in microarray design, investigators and analysis methods. This analysis also expanded the “ethanol responsome” nearly 10-fold. VMB exhibited the largest transcriptional response to ethanol, while changes observed in PFC and NAc were of comparable magnitude (Figure 1a). The transcriptional response to ethanol within each brain region included both unique and shared gene components. Roughly 1/3 of significantly ethanol-responsive genes in the PFC and NAc were unique to their respective regions, while greater than 50% of the VMB ethanol profile was specific to that region (Figure 1b).

Functional enrichment analysis showed strong homology in the functional categories regulated by ethanol in all three regions (Table S2). Gene groups related to synaptic activity and plasticity were among the most significantly over-represented GO biological functions, with dendritic or synaptic structure as the top GO cellular components in each region. The 399 genes that were significantly ethanol-responsive in all three brain regions were also highly enriched for proteins that localize to the pre- and post-synaptic membranes and regulate synaptic transmission, including both ionotropic and metabotropic glutamate receptor categories (Table S2). However, there were regional differences, the over-representation of GABA and glutamate receptor signaling pathways was particularly high in VMB.

**Figure 2:**
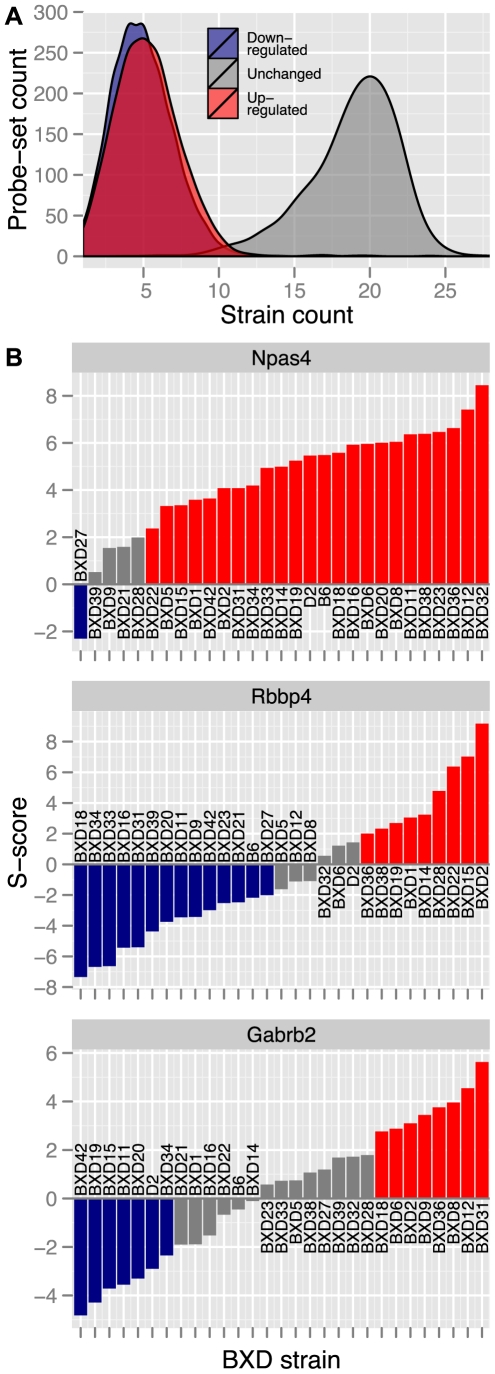
Acute ethanol transcriptional response profiles. (**A**) Strain frequency distributions of gene transcriptional-response classes based on PFC S-score analysis. S-scores >2 indicate a gene was up-regulated by acute ethanol, S-scores <2 indicate down-regulation and S-scores between these thresholds were considered unchanged. (**B**) S-score strain distributions for three significantly ethanol responsive genes that each represent a different class of ethanol response profile.

Assaying gene expression across the BXD panel allowed us to analyze how genetic variation influenced transcriptional responses to ethanol (Figure 2). As seen with other heritable complex traits measured in genetic mapping panels, the transcript-level response of most ethanol sensitive genes followed a continuous distribution across the BXD and progenitor strains. There was a subset of genes almost uniformly up-regulated by ethanol, including *Npas4* (Figure 2b), *Fos*, *Hsp8*, *Egr2*, *Dusp1* and *Jun*, all of which are neuronal activity dependent. Most genes, however, exhibited divergent ethanol responses between variable subsets of BXD strains (Figure 2a). While continuous distributions of transcriptional responses to ethanol were observed in all profiled regions, transcript-level changes were highly region specific. That is, we found little correspondence between a gene's ethanol response across regions. Even among the 399 genes found to be significantly ethanol responsive in all three brain regions, inter-region S-score correlations were effectively null (Figure S2). Therefore, acute ethanol effects on gene expression were modulated by both genetic background and brain regional environment factors.

### Gene network analysis in prefrontal cortex

Rather than focusing on gene-lists, as was only possible in our prior analysis limited to the B6 and D2 strains, we used the power of genetic correlations across the BXD strains to derive coherent gene networks. Due to the complexity of this analysis and the importance of the PFC in influencing long-term adaptive responses to ethanol and goal-directed behavior [44], [45], [46], we restricted our network analysis to this brain region. A detailed analysis of network interactions across PFC, NAc and VMB will be presented elsewhere (Wolen and Miles, manuscript in preparation).

**Figure 3:**
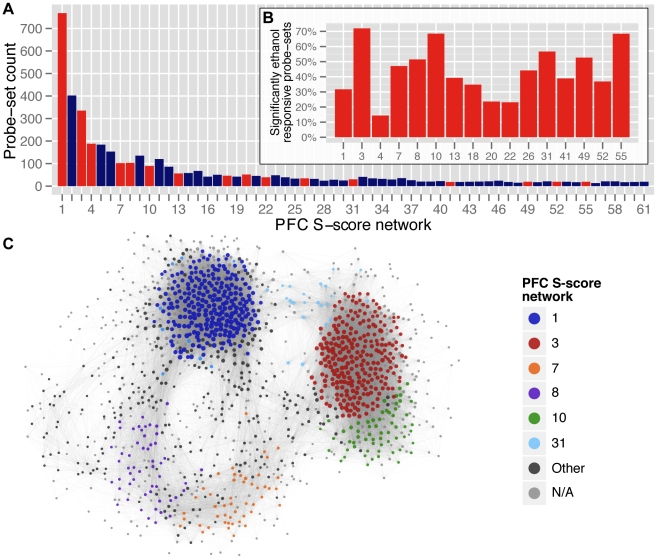
Saline vs ethanol S-score paraclique networks. (**A**) Distribution of S-score network sizes based on the number of genes assigned to each. Significantly ethanol-responsive genes were over-represented in a subset of these networks (red bars). (**B**) These 16 paracliques were considered ethanol-responsive gene-enriched networks (*ErGeNs*). (**C**) Network-based clustering of the 1,246 significantly ethanol-responsive genes in the PFC revealed distinct modules largely corresponding to the ErGeNs depicted in B.

We studied saline vs ethanol S-score expression data, as well as individual saline- and ethanol-treatment RMA expression data, using a graph theoretical algorithm that identified discreet paracliques within each dataset [32]. As described in Methods, the resulting paracliques, referred to as networks henceforth, represent densely intercorrelated groups of genes that likely share functional and regulatory homology. In the saline and ethanol networks formed with RMA expression datasets, inter-gene correlations represented the admixture of treatment variation superimposed on basal steady-state mRNA levels. In the context of the S-score networks, the correlations strictly reflect coordinated changes in expression induced by acute ethanol. The size of these networks ranged from 710 to 11 probe-sets (Figure 3a; Table S3).

While 64% of all significantly ethanol-responsive genes in the PFC belonged to one of the 61 S-score networks, a Fisher's exact test revealed a subset of networks that were statistically enriched for these genes. These ethanol-responsive gene enriched networks (ErGeNs) are depicted in Figure 3b. Network-based clustering (Figure 3c) and a traditional non-parametric partitioning (Figure S3) of all significantly ethanol responsive genes, both revealed the existence of several modules of co-expressed genes that were largely subcomponents of these paraclique-derived *ErGeNs*, most predominantly *ErGeN1* and *ErGeN3*. Taken together, these results suggested that, at the time point employed by these studies, the PFC transcriptional response to acute ethanol was primarily mediated through a relatively small number of highly organized gene networks.

To determine how networks generated from the different treatment groups (saline vs ethanol networks) and analyses (saline/ethanol networks vs S-score networks) related to each other, we performed pairwise comparisons of all network members (Figure S4; Table S4). Many of the saline networks significantly overlapped with networks in the ethanol data, indicating the inter-gene correlations that constitute these networks are largely stable across treatments and likely represent robust biological relationships. Similarly, S-score networks generally had a substantial and predominant relationship with a single or small number of saline or ethanol networks, as might reflect the contribution of basal expression levels and the mathematical derivation of S-scores from saline and ethanol expression data.

**Figure 4:**
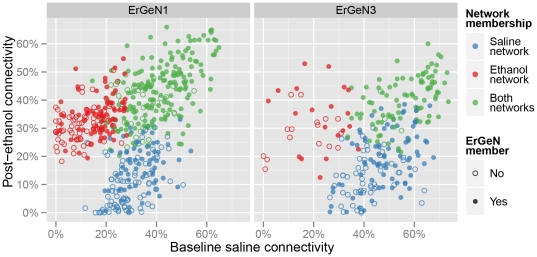
Relationship between ErGeNs and counterpart networks in RMA expression data. Both S-score networks, ErGeN1 and ErGeN2, had counterpart networks in the basal saline and post-ethanol expression data: ErGeN1 significantly overlapped with saline network 1 and ethanol network 1; ErGeN3 significantly overlapped with saline network 4 and ethanol network 2 (Figure S3). Each point represents a gene that belongs to a given ErGeN's counterpart saline network (blue), ethanol network (red) or both (green). Filled-in points indicate the gene also belongs to the overlapping ErGeN. The X- and Y-axes measure gene connectivity (|Pearson correlation coefficient|≥0.7) within the saline and ethanol expression datasets, respectively.

We examined in detail how the two major networks comprising the PFC transcriptional response to ethanol, ErGeN1 and ErGeN3, related to their respective counterparts in the saline and ethanol RMA expression data, in order to determine what additional information is provided by the S-score networks. ErGeN1 was significantly enriched for members of saline network 1 and ethanol network 1. Likewise, the gene members of saline network 1 and ethanol network 1 significantly overlapped each other, with 215 genes in common. The overlapping components of these three networks were frequently the mostly highly connected nodes (Figure 4, ErGeN1 panel). ErGeN3 exhibited a similar relationship with saline network 4 and ethanol network (Figure 4, ErGeN3 panel). Therefore, these S-score networks largely comprise the robustly inter-connected hubs of existing networks. However, missing from Figure 4 are the 439 and 143 probe-sets that belong to ErGeN1 and ErGeN3, respectively, but not their counterpart networks in the saline or ethanol RMA expression data. These network facets unique to the ErGeNs represent a form of genetic co-regulation that would have gone undetected without the use of S-score data.

### Genetic regulation of ethanol-responsive networks

To uncover the genetic elements co-regulating these networks we performed expression QTL (eQTL) mapping for each probe-set's expression trait in the saline and S-score data (Table S5). Doing so across both datasets allowed us to assess how the baseline regulatory architecture of the PFC transcriptome is altered by exposure to acute ethanol. Interestingly, the genetic regulatory profiles for the two datasets differed substantially. Although the majority of probe-sets mapped to at least one suggestive eQTL (Table 1), only 6% of eQTL positions were conserved in both the saline and S-score datasets. Indeed, we observed a fundamental shift in the type of genetic regulation most prominent across these datasets. Of the 3,279 genes with significant eQTL in the saline expression data, 42% were considered to be *cis*-acting, since the peak eQTL location mapped within 5 Mb of the linked expression trait. Whereas in the S-score data *cis* eQTL accounted for less than 1% of the 1,215 genes with significant eQTL.

**Table 1:** Expression QTL mapping results for saline RMA and S-score datasets.

Dataset	eQTL class	Suggestive eQTL (# genes)	Significant eQTL (# genes)
**Saline**	*trans*	9,570	1,877
	*cis*	433	1,355
**S-scores**	*trans*	10,968	1,276
	*cis*	62	7

P-value thresholds are genome-wide corrected with cutoff values defined as in Methods.

The effective absence of *cis* eQTL in the S-score data suggests that mechanisms underlying ethanol-responsive gene regulation may fundamentally differ from those governing basal transcription. However, some portion of the basal *cis* eQTL are likely spurious associations driven by polymorphisms between the B6 and D2 genomes (see Table S8) that affect microarray probe target hybridization [47], [48]. As the impact of such SNP effects should be invariant across the saline vs. ethanol treatment conditions, any spurious *cis* eQTL would be effectively filtered out of the S-score eQTL results.

**Figure 5:**
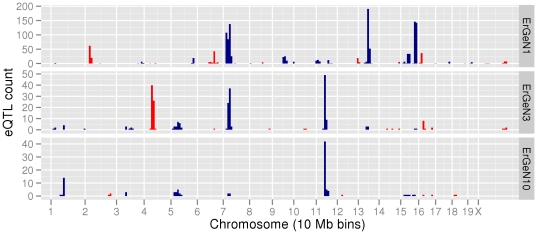
Genetic regulatory architecture of major ErGeNs. Histogram of saline vs ethanol S-score eQTL (genome-wide p-value<0.63) frequencies across the genome divided into 10 megabase (Mb) bins.

Similar to other genetical genomics studies, we found that many changes in transcript abundance induced by acute ethanol were linked to a relatively small number of highly influential loci, so-called ‘regulatory hotspots’ or trans-bands. This was particularly salient for eQTL profiles of the major ErGeNs (Figure 5). These networks could largely be partitioned into 6 trans-bands that mapped to loci on Chr 4, 7, 11, 13, 15 and 19. In most cases, these trans-bands were unique to specific networks, the exceptions being the Chr 7 and Chr 11 trans-bands, which were composed of genes from *ErGeN1* & *ErGeN3*, and *ErGeN3* & *ErGeN10*, respectively (Table 2).

**Table 2:** ErGeN trans-band locations.

Chr	ErGeN ID	Peak marker position (Mb)	Support interval (Mb)
4	ErGen3	46.61 (rs13477694)	35.49–55.07
7	ErGen1 ErGeN3	34.62 (rs3694031) 30.14 (rs8261944)	15.52–36.48 24.06–30.43
11	ErGeN3 ErGeN10	58.38 (rs3697686)	53.89–68.93 56.35–62.07
13	ErGeN1	54.88 (rs13481817)	47.68–69.04
15	ErGeN1	89.87 (rs13482702)	86.80–95.78
19	ErGeN7	41.69 (rs3653396)	32.73–41.95

### Hub genes

The parameters used to construct the networks described above were such that the vast majority of genes share edges with at least half of the remaining network. Subsets of genes shared edges with nearly all network members, and were more important to the network based on measurements of connectivity and centrality. These network hub genes could be major regulators of the transcriptional response to acute ethanol and more generally, may represent key points of vulnerability in underlying signaling pathways responding to ethanol. We therefore identified hub genes by ranking network members based on their degree of connectivity and betweenness centrality. (Table S3).

**Figure 6:**
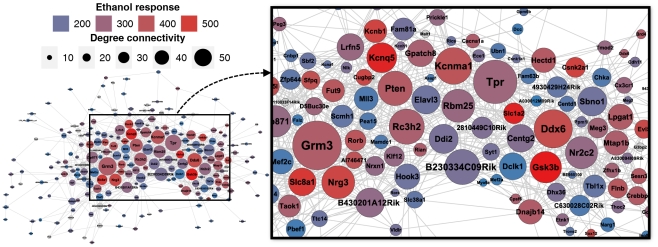
Hub genes within ErGeN3. Network visualization of all genes comprising ErGeN3 that share at least one adjacent edge at a correlation threshold of >|0.9|. Node color indicates the magnitude of a gene's transcriptional response to ethanol, quantified using Fisher's combined p-values. Grey nodes were not altered by ethanol. Node size represents a genes degree of connectivity.

Among the most highly connected hubs within *ErGeN3* were a number of genes that have been previously implicated in modulating level of response to ethanol or susceptibility to alcohol dependence (Figure 6), including *Kcnma1* and *Gsk3β*. *Kcnma1* is a large conductance potassium channel whose activity is directly affected by ethanol [49]. *Gsk3β*, is a serine/threonine kinase that participates in the WNT signaling pathway and is an important modulator of ethanol-induced neurotoxicity in both mice [50] and Drosophila [51]. These findings on *Kcnma1* and *Gsk3β* serve to validate our network analysis approach, identifying these and other hub genes (Figure 6) as potentially important modulators of ethanol phenotypes.

The *ErGeN3* member with the highest degree of connectivity was a probe-set (1435583_at) annotated as *AU067633*. However, recent data from RNA-Seq analysis of B6 and D2 brain transcripts (Lu and Williams, personal communication) strongly suggests that this probe-set actually targets the distal 3′ untranslated region of *Grm3*, a metabotropic glutamate receptor (Figure S5). Given the considerable evidence that metabotropic glutamate receptors are key mediators of the neuroadaptations associated with addiction [52], *Grm3*'s position as a major hub of this ethanol-responsive network has mechanistic implications for regulation of the network and further supports the overall significance of this network in ethanol traits.

### Candidate regulators of ethanol-responsive networks

We sought to identify candidate regulators of ErGeNs by dissecting the hotspots underlying each network trans-band. Positional candidate genes located within trans-band support intervals were empirically ranked using an integrative strategy that combined DNA sequence polymorphisms and results from the differential expression, eQTL and network connectivity analyses. The full list of ranked candidate genes for each trans-band is provided in Table S6.

The two largest ethanol-responsive networks, *ErGeN1* and *ErGeN3*, shared a common regulator on the proximal end of Chr 7, between 15.52 and 36.48 Mb (Table 2). Examination of eQTL for all members of these networks revealed a complicated pattern of association, in which the trans-band could be subdivided into several groups based on peak eQTL locations that clustered between 16.3 and 35.04 Mb (Figure S6). Peak linkage of genes from *ErGeN3*, however, was limited to a narrow region between 30.1 and 30.2 Mb, at the distal edge of the support interval. This locus represents the common regulatory hot-spot shared by these two networks and harbors the two most highly ranked candidate regulators of the Chr 7 trans-band: *Scn1b*, a voltage gated sodium channel subunit and *Aplp1*, amyloid beta precursor-like protein (Table 3). Both genes were significantly ethanol-responsive, highly connected hub nodes in *ErGeN1* and associated with cis eQTL in the saline data. Unlike *Aplp1*, The ethanol response of *Scn1b* was at least partially regulated by a local polymorphism, as evidenced by its suggestive cis eQTL in the S-score data. Both genes contain coding polymorphisms, *Aplp1*'s harbored a polymorphic splice site, raising the possibility that different *Aplp1* isoforms may segregate members of the BXD family.

**Table 3:** Candidate genes in ErGeN trans-band support intervals.

Trans-band	Gene	ErGeN	Diff. exp. q-value	Network scaled		cis eQTL p-value		SNPs	
				connect.^a^	centrality	saline	s-score	coding	ns^b^
Chr 7	Aplp1	1	0.04	0.95	0.92	0.004		6	3
Chr 7	Scn1b	1	0.02	0.94	0.75	0.05	0.51	6	1
Chr 11	Gria1	3	0.03	0.49	0.56	2×10^−5^		16	0
Chr 11	Ncor1	10	0.005	0.03	0.75		0.19	14	5
Chr 13	Sncb	1	0.008	0.98	0.92	0.2	0.21	1	0
Chr 15	Nell2	1	0.002	0.94	0.99	0.01		4	2

Differential expression q-values and network parameters defined in Methods.

a Connectivity;

b Non-synonymous SNPs.

Of the *ErGeN1* genes without a *trans* eQTL on proximal Chr 7, most could be partitioned into trans-bands linked to Chr 13 or 15. The regulatory hotspots underlying these trans-bands were both unique to *ErGeN1* (Table 2). The Chr 13 trans-band support interval spanned from 47.6 to 69 Mb and peaked at 54.88 Mb. QTL for both cocaine induced activation [53] and hypothalamic corticotropin-releasing factor binding protein (*Crf-BP*) transcript abundance [54] were previously mapped to this region. Ranking the positional candidates within this region revealed a promising candidate in *Sncb* (Synuclein beta), a neuronal protein that is widely co-localized to presynaptic terminals throughout the brain [55]. *Sncb* was one of the largest *ErGeN1* hub genes and was regulated by suggestive *cis* eQTL in both the saline and S-score datasets.

The regulatory hotspot underlying the Chr 15 trans-band has previously been implicated as a regulator of two ethanol behavioral phenotypes, including an ethanol preference QTL mapped using congenic lines derived from B6 and BALB/cJ mice [*56*]; as well as a QTL underlying loss of righting due to ethanol [*57*], [*58*]. The primary candidate regulator of this trans-band was *Nell2* (Protein kinase C binding protein), which showed the highest regional response to ethanol. Nell2 was an important hub of *ErGeN1*, as the network's fifth most central gene. While *Nell2*'s baseline transcription was strongly regulated by a *cis* eQTL, its ethanol response was modulated by the Chr 13 regulatory hotspot.

Similar to the Chr 7 trans-band, the regulatory hotspot on Chr 11 was linked to trans-bands from multiple networks, *ErGeN3* and *ErGeN10* (Table 2). Two strong candidate genes emerged from this region: *Gria1* and *Ncor1* (Table 3). From a hypothetical functional perspective, both genes are highly intriguing candidates; *Gria1*, as an ionotropic glutamate receptor and *Ncor1* as a transcriptional repressor acting through nuclear receptors and histone deacetylation. In our expression data, both genes were significantly ethanol-responsive, however, *Ncor1*'s response was stronger than *Gria1*'s. Furthermore, while the baseline expression of *Gria1* was primarily regulated by a highly significant *cis* eQTL, regulation of *Ncor1* was modulated by a suggestive *cis* and *trans* eQTL, the latter of which coincided with the Chr 7 trans-band. Reanalysis of Ncor1's expression using a two-locus model revealed a significant interaction between the Chr 11 and Chr 7 eQTL (data not shown).

### Biological relevance of ethanol-responsive networks

As done for total ethanol-responsive gene sets, we investigated GO or pathway functional over-representation for the S-score networks. The vast majority of networks were over-represented for at least one gene family, protein domain/interaction, KEGG pathway or GO category, significant at a FDR level of 5% (Table S7). *ErGeN1* was strikingly enriched for proteins with GTPase activity (p-value = 1.5E-07), including *Rab3a*, which mediates ataxic consequences of ethanol consumption and influences ethanol preference [59]. Both *ErGeN1* and *ErGeN3* were significantly enriched for genes encoding proteins that localize to the synapse (Table 4). In contrast, S-score networks 2 and 12 had a large over-representation of genes related to ribosome function and oxidative phosphorylation.

**Table 4:** Functional analysis of major ErGeNs.

Functional category	Source	FDR p-value	# of genes
**ErGeN1**			
GTPase activity	GO:MF	1.5E-07	26/219
Regulation of synaptic transmission	GO:BP	1.85E-07	21/153
Neurotransmitter secretion	GO:BP	2.31E-06	14/85
Synapse part	GO:CC	3.08E-09	32/270
Dendrite	GO:CC	8.56E-09	25/182
Synaptosome	GO:CC	7.85E-07	15/91
PTEN pathway	MigDB	2.86E-06	7/18
**ErGeN3**			
RING-type zinc fingers	HGNC	1.2E-07	16/209
Synapse part	GO:BP	1.83E-06	16/270
FHF complex	GO:CC	2.84E-05	3/5
Histone deacetylase complex	GO:CC	3.56E-04	5/43
Potassium channels	HGNC	2.28E-05	6/88

**Figure 7:**
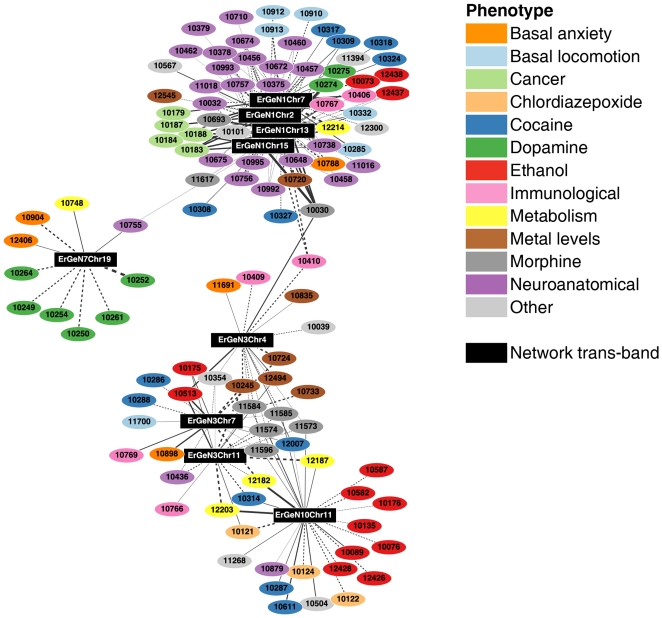
ErGeN trans-bands have distinct phenotypic correlations. Correlations between principal component traits of ErGeN trans-bands and BXD phenotypes (p-value<0.01). Edge thickness indicates strength of network/phenotype association and dashed lines indicate a negative correlation. Each phenotype node is labeled with a trait ID that can be queried on GeneNetwork.

Using the BXD panel of mouse strains also allowed for direct comparison of ethanol gene expression data with the wide variety of phenotypic traits previously profiled in the BXD strains. To detect high-level phenotypes regulated by ethanol-responsive gene networks, we tested associations between *ErGeN*s and over 2,000 phenotypes available from GeneNetwork. This analysis was conducted by measuring correlations between GeneNetwork phenotypes and synthetic traits generated by principal component analysis of *ErGeN* trans-bands (Figure 7). The first principal component of each trans-band was used for computational ease and clarity. Performing this analysis at the network and trans-band level made it possible to detect patterns of phenotypic associations with improved specificity. As expected, the analysis showed a striking clustering of trans-bands for individual ErGeNs and associated phenotypes.

The GeneNetwork phenome database contains a large number of neuroanatomical morphometric measurements. Many of these [60] were strongly associated with *ErGeN*1 in its entirety (i.e., all trans-bands), including ventral hippocampus volume, overall brain weight, dorsal thalamus volume and amygdala basolateral complex volume [54]. This network was also highly correlated with the *B*_max_ for naloxone binding [61], a μ-opioid receptor antagonist that is an approved treatment for alcoholism. Whereas only a subset of *ErGeN1*'s trans-bands were correlated with morphine metabolism rate [62]; the same two trans-bands also correlated with ethanol acceptance in a two-bottle choice test [63].

This analysis also revealed *ErGeN*'3s to be important potential mediators of phenotypic responses to several drugs of abuse. As a whole, *ErGeN1* impacts both baseline locomotor activity [64] and habituation [65] in novel open field tests, but the effect of cocaine on these phenotypes was primarily correlated with *ErGeN1*'s Chr 7 trans-band. Interestingly, non-locomotor based responses to cocaine were associated exclusively with *ErGeN3*, including measurements of stereotypic repeated movements [66], [65] and conditioned place preference for the drug (Phillips et al., unpublished). Given the importance of dopamine levels in activating these behaviors, particularly stereotypy, we expected to find a strong connection between *ErGeN1*'s Chr 7 trans-band and the dopamine binding phenotypes included in the GeneNetwork database. Instead, we observed that *ErGeN7*'s solitary trans-band on Chr 19 to be the primary correlate of these measurements, which included *Drd1* & *Drd2* binding density in the dorsal striatum and NAc [65].

Along with *ErGeN1*, *ErGeN3* was related to Naloxone *B*_max_ concentration but also showed strong correlations with morphine induced locomotor activation and naloxone induced morphine withdrawal (Phillips et al. unpublished). These morphine phenotypes were also connected to *ErGeN10*. This overlap is perhaps not surprising given the strong association between many genes within *ErGeN3* and *ErGeN10* (Figure 3c), as well as the shared trans-band support interval on Chr 11. However, one distinction between *ErGeN3* and *ErGeN10* was the clustered connections of numerous ethanol relevant phenotypes to *ErGeN10*. While *ErGeN3* correlated with ethanol metabolism rate [67] and blood glucose levels following ethanol treatment [68], *ErGeN10* appears more related to ethanol behavioral phenotypes, including ethanol induced locomotor activation [63], anxiolysis (Cook et al., unpublished) and sensitization [69].

## Discussion

Here we have presented results from the first genetic analysis of acute ethanol-responsive gene expression in the three major brain regions comprising the mesocorticolimbic reward pathway, and a comprehensive characterization of gene networks that constitute this gene expression response in PFC. Our analysis identified unique gene networks with implications on ethanol-evoked neuroadaptive mechanisms and behaviors, and showed that the response of such networks is governed by overlapping sets of discreet genetic loci. Perhaps most importantly, this analysis highlighted a series of hub genes as potentially major factors influencing brain responses to ethanol, setting the stage for future mechanistic studies and possible development of novel therapeutic approaches to alcoholism.

The approach used here to identify ethanol-responsive genes was somewhat unorthodox for a microarray study. Rather than comparing two treatment groups composed of multiple biological replicates, our treatment groups comprised relatively large samples of 29–36 genetically unique individual strains. Although only single arrays were performed per strain/treatment, the issue of biological variability was reduced by pooling tissue samples from at least 4 biological replicates per strain. Our use of the S-score analysis method to compare ethanol vs. saline responses further improved the robustness of our genetic correlation analysis [70]. Gene expression correlations or expression-genotype correlation significance were also empowered by the number of strains compared. The approach identified a robust set of genes whose expression levels were significantly altered by ethanol across the BXD strains in at least one of the three profiled brain regions. This gene set included a large contingent of our prior 2-group microarray study of brain ethanol-responsive genes [21]. Strikingly, nearly a quarter of the ethanol-responsive genes defined here were previously identified as having basal expression differences in a meta-analysis by Mulligan et al. [71] of microarray data from whole brain RNA of a number of inbred lines selected for divergent ethanol preferences (Figure S7). It's likely the extent of this overlap would have been greater if that meta-analysis had been conducted across targeted brain regions, rather than whole brain. Regardless, many of the genes whose basal expression levels segregate with alleles driving divergent preferences for ethanol were also regulated upon exposure to acute ethanol in our study. This finding both adds validation to both studies and further emphasizes the relevance of studying acute molecular or behavioral responses to ethanol in terms of their implications for molecular events underlying chronic ethanol behaviors.

One potential confound in our analysis of ethanol-responsive gene networks regards the experimental design used for microarray studies. Since the BXD strains used for tissue harvesting were also part of a behavioral genetics analysis on ethanol anxiolytic actions (Putman, Wolen and Miles, manuscript in preparation), the animals received mild restraint stress and behavioral testing, in addition to saline or ethanol treatment (see Methods). Use of S-scores to compare saline vs. ethanol-treated animals was calculated to remove the effect of stress from the derived expression patterns since both groups were handled identically. However, we cannot rule out that an interaction between stress and ethanol, rather than just a response to acute ethanol, might contribute to some of the gene networks found in our studies. Regardless, the large overlap between expression patterns derived here for acute ethanol and a published study on basal gene expression correlating with predisposition to ethanol consumption [71], does lend strong support to the argument that the networks discussed here are likely to be important in behavioral responses to ethanol.

Additional technical factors to be considered in these studies regards the use of only a single microarray per strain/treatment group and the possibility of SNPs affecting microarray probe performance. Although arrays were derived from pooling tissue across 4–5 animals, technical variance could have influenced our results. We believe that such variance would have likely only degraded expression correlations and done so partially given the number of strains used for the genetic correlation analysis. In particular, identification of overlapping trans-eQTL for many genes within a given paraclique (Figs. 5 and S6) is strong evidence for the technical rigor of these studies since such genetic correlations would have been severely affected by technical variance. Regarding potential SNPs affecting hybridization results, this issue was discussed in Methods and a complete list of SNPs identified in probes is included in Table S8. Since our analysis largely focused on ethanol-regulated gene expression and the S-score analysis would cancel out any SNP effect (since both control and ethanol treated samples would be affected), we chose not to eliminate SNP containing probes from our analysis but did penalize them during candidate gene ranking (see Methods).

This genetic analysis of ethanol-responsive gene expression allowed extension beyond dichotomous gene lists, to the spectrum of acute ethanol transcriptional responses influenced by naturally occurring polymorphisms segregating in the BXD strains. This approach identified gene groups having by a wide range of differential expression profiles: including genes such as *Npas4*, which was consistently up-regulated by ethanol, and *Gabrb2*, whose response entailed up-regulation, down-regulation and no change, depending on the subset of strains (Figure 2). Such a range of expression changes highlights the complex role of genetic background in modifying molecular responses to acute ethanol exposure.

We leveraged the genetic variance in ethanol expression profiles by deriving dense paraclique gene networks co-regulated by acute ethanol. These networks likely represent initial perturbations of key molecular pathways, which, upon repeated consumption of ethanol, produce downstream neuroadaptations associated with alcohol abuse and dependence. The functional results of *ErGeN1* and *ErGeN3* (both of which were highly populated with robust ethanol-responsive genes) support this assertion, as both networks were significantly enriched for proteins involved in neurotransmission and synaptic plasticity (Table 4, Table S7).

A valuable advantage of such network-based approaches is that the relative importance of specific genes can be assessed in part, by the context of their surrounding interactions. There is a continuously growing body of evidence suggesting that hub genes are of particular importance to genetic networks. For example, introducing null mutations into hub genes negatively impacted the hardiness of *Escherichia coli* to a much greater extent than did mutations of randomly selected genes [72]. This may be explained by an observation made in *Caenorhabditis elegans*, showing that hub genes participated in a variety of canonical signaling pathways [73]. In a genetic network study of mouse liver, hypothalamus and adipose tissue, hub genes were also found to be highly connected nodes across all three expression datasets [74]. In the results presented here, we too found that many hub genes in basal networks maintained hub status within *ErGeN*'s as well (Figures 4 and 6).

The major hub genes of PFC saline vs ethanol S-score networks, and particularly *ErGeN3*, included a number of genes previously implicated in drug dependence and neurological disease. The aforementioned node with the highest betweenness centrality in *ErGeN3* was a probe-set targeting *Grm3*. It is well established that metabotropic glutamate receptors play an important functional role in the development of AUD [75], [52], [76]. Studies have demonstrated, in particular, that modulation of *Grm3* decreases ethanol seeking in rats [77], [78]; although the agonists used in these studies also bind *Grm2*. *Grm3* is also a high priority candidate gene for schizophrenia, as a group II mGluR agonist (LY354740) blocked many symptoms induced in the rat phencyclidine treatment model of schizophrenia [79]. *Grm3* has also been associated with schizophrenia phenotypes in human genome-wide association (GWA) studies [80]. Among the genes adjacent to *Grm3* in *ErGeN3*, the strongest correlation was between *Grm3* and *Nrg3* (r = 0.97, p-value<1e-16). Like *Grm3*, *Nrg3* (neuregulin 3) is a highly connected gene in *ErGeN3* as well as a schizophrenia candidate gene [81], [82].

The large conductance potassium channel, *Kcnma1*, was also an *ErGeN3* hub gene (Figure 6). In addition to its known functional response to ethanol exposure [49], *Kcnma1* is a very intriguing hub gene because it is a proven major regulator of acute ethanol-induced intoxication in *C. elegans* [83]. Furthermore, two recently published human GWA studies have provided preliminary evidence for a link between *Kcnma1* and alcohol dependence [84], [85]. The study by Kendler and colleagues also identified another voltage gated potassium channel, *Kcnq5*, as having an association with AD. This is an exciting result, as *Kcnq5* is directly adjacent to *Kcnma1* in *ErGeN3*, and both genes are highly ethanol-responsive and major hubs of the network.

In addition to identifying hub genes as leading candidates for future verification studies, our genetic dissection of ethanol-responsive gene networks also produced clues regarding the mechanisms underlying ethanol network responses. Identification of chromosomal hot spots linked to ethanol responses for entire gene networks provides genetic evidence for hubs influencing the response of *ErGeN*'s and expands our understanding of brain molecular signaling events responding to ethanol. For example, the sodium channel *Scn1b* was a hub gene in *ErGeN1*, showed robust ethanol-responsiveness, had a highly significant cis-eQTL and also was a strong candidate for regulating a trans-band of *ErGeN3* mapping to exactly the location of *Scn1b*. *Scn1b* codes for a regulatory subunit of sodium channels which are crucial to action potential propagation. Ethanol has been shown previously to inhibit sodium channel function [86]. This data suggests that *Scn1b* and other such potential regulators of ethanol-responsive trans-bands may be key modulators for extensive portions of the overall ethanol responsome.

Defining complex endophenotypes such as acute ethanol sensitivity in terms of gene networks, rather than the genetic variants that influence them, has the potential to yield information about complex diseases that is more generalizable to humans. Network function, rather than individual gene influences, is likely more conserved evolutionarily. The ethanol-responsive gene-enriched networks defined here could assist human GWA studies by providing a novel source of functionally related candidate genes. As mentioned above, the fact that several of the major *ErGeN* hub genes have been recently implicated in GWA studies suggests this approach is highly promising.

Co-analysis of human GWA studies and *ErGeN* hub genes may provide a bidirectional validation for such genes, even leading to candidates for therapeutic targeting. However, taken out of context, such single genes still do not define the mechanisms underlying cellular, neural network or behavioral responses to ethanol, which remains our chief objective in identifying and dissecting these gene networks. Direct validation of hub genes, in terms of both gene network regulation and phenotypic responses, are required to fully understand the role of these ethanol-responsive networks in complex behavioral responses. Ongoing studies in our laboratory seek to adapt and extend this approach, through genetic manipulation of *ErGeN* hub genes, in order to observe downstream effects on the original ethanol-responsive network as well as the network-associated ethanol behavioral phenotypes. Such validation of network-derived candidates could provide a novel approach to future pharmacotherapies for AUD, directed against regulation of a gene network rather than function of a single protein.

## Supporting Information

Figure S1 **Outline of integrative strategy used to prioritize positional candidate genes underlying ErGeN trans-bands.**
(TIFF)

Figure S2 **In order to determine the degree to which a gene's transcriptional response to ethanol is tissue-specific, we calculated cross-regional S-score correlations for each of the 399 probe-sets that were significantly ethanol responsive in the PFC, NAc and VMB.** (**A**) The distribution of Pearson correlation coefficients and (**B**) corresponding p-values, indicate there is effectively no coordinated response to ethanol across regions.
(TIF)

Figure S3 **Traditional non-parametric partitioning and clustering of all significantly ethanol-responsive genes in the PFC.** The number of modules was determined by principal component analysis, which revealed the first 4 components explained ∼70% of the variation in these genes S-scores (**A**). Genes were assigned to modules by partitioning around medoids, which were then independently hierarchically clustered based on average linkage of Pearson correlations. These results are visualized in the heatmap (**B**). Warmer colors represent positive S-scores (up-regulated by ethanol) whereas cooler colors indicate negative S-scores (down-regulated by ethanol). The adjacent column of colors indicates to which PFC S-score network the corresponding gene was assigned.
(TIFF)

Figure S4 **Substantial overlap existed between the gene constituents of paraclique networks formed with the saline (blue squares) and ethanol (red diamonds) RMA expression datasets, and the saline vs ethanol S-score (green circles) expression data.** The statistical significance of overlap between networks was determined by Fisher's exact test, which identified 161 inter-dataset networks with more genes in common (edge numbers) than would be expected by chance, based on a Bonferroni-corrected p-value<0.05 (Table S4). The figure depicts a subset of the overlapping inter-dataset network relationships that share at least 15 genes in common. Each shape represents the co-expression network specified by its label. Node color and shape indicate the expression dataset used to form the network, while node size is proportional to the number of genes comprising the network. Edge thickness represents the statistical significance of the overlap.
(TIF)

Figure S5 **Whole brain RNA-Seq expression data across the Chr 5 region that encompasses** ***AU067633*** **and** ***Grm3*** **(A), adapted from GeneNetwork mirror of the UCSC Genome Browser (ucscbrowser.genenetwork.org).** Although probe-set 1435583_at (red) putatively maps to an *AU067633* intron, it appears to actually target *Grm3*'s 3′ UTR, which is highly expressed from the negative strand across the same stretch of DNA. Probe-set 1435583_at's basal RMA expression levels were significantly correlated with the distal *Grm3* probe-set, 1430136_at, while showing no relationship to the proximal *AU067633* probe-set, 14338324_at (**B**).
(TIFF)

Figure S6 **Support intervals for the major eQTL hotspot on Chr 7 for** ***ErGeN1*** **(A) and** ***ErGeN3*** **(B), and the eQTL hotspot on Chr 11 for** ***ErGeN3*** **(C) and** ***ErGeN10*** **(D).** Each horizontal line represents an individual probe-set's 1.5 LOD drop support interval, ordered and colored based on peak LOD score. Blue ticks indicate peak eQTL locations. The heatmap along the x-axis represents the percentage of probe-set support intervals that encompass the underlying markers.
(TIFF)

Figure S7 (**A**) Overlap between 2,743 genes that exhibited a significant response to acute ethanol across the BXD family in PFC, NAc or VMB and 3,859 genes identified as differentially expressed between several high and low ethanol preferring strains in a meta-analysis of whole brain tissue [70]. Overlap significance was measured using a one-tailed Fisher's exact test. (**B**) Distribution of the intersecting acute-ethanol/ethanol-preference genes among profiled regions of mesocorticolimbic CNS reward circuit.
(TIFF)

Table S1 **Regional Differential Expression Results.** (**A**) Affymetrix probeset IDs, gene symbols, Entrez gene identifiers and differential expression q-values for genes significantly regulated by ethanol in PFC across BXD strains. (**B**) Values for NAc. (**C**) Values for VMB.
(XLS)

Table S2 **Results for functional over-representation analysis of genes differentially expressed in PFC (A), NAc (B),VMB (C) and intersection across regions (D).**
(XLS)

Table S3 **PFC paraclique networks.** Gene identifiers and network parameters (connectivity and centrality) for Saline (A) versus S-score (B) paracliques.
(XLS)

Table S4 **Overlapping cross treatment paraclique networks.** Matching of paraclique networks across saline, ethanol or S-score datasets from PFC.
(XLS)

Table S5 **Expression QTL analysis of PFC paraclique networks.** Peak QTL positions and significance are indicated for genes from saline (A) versus S-score (B) paraclique analyses.
(XLS)

Table S6 **Ranked trans-band positional candidate genes.** Results of empirical ranking scheme for saline versus S-scores paracliques are shown for eQTL mapping to Chr 4, 7,11, 13, 15 and 19.
(XLS)

Table S7 **Functional analysis of PFC paraclique networks.** Data is from ToppGene functional over-representation analysis of paraclique networks.
(XLS)

Table S8 **D2 SNPs overlapping Affymetrix Mouse 430 type 2 microarray probes.** Table indicates location and sequence of SNPs existing between B6 versus D2 mice and the position of probeset sequences from the Affymetrix 430 type 2 arrays.
(XLS)
