# Supplementary figures and images for: Genetic Dissection of Acute Ethanol Responsive Gene Networks in Prefrontal Cortex: Functional and Mechanistic Implications
Source: PLoS One. 2012 Apr 12;7(4):e33575. doi: 10.1371/journal.pone.0033575 (PMC3325236; doi:10.1371/journal.pone.0033575)

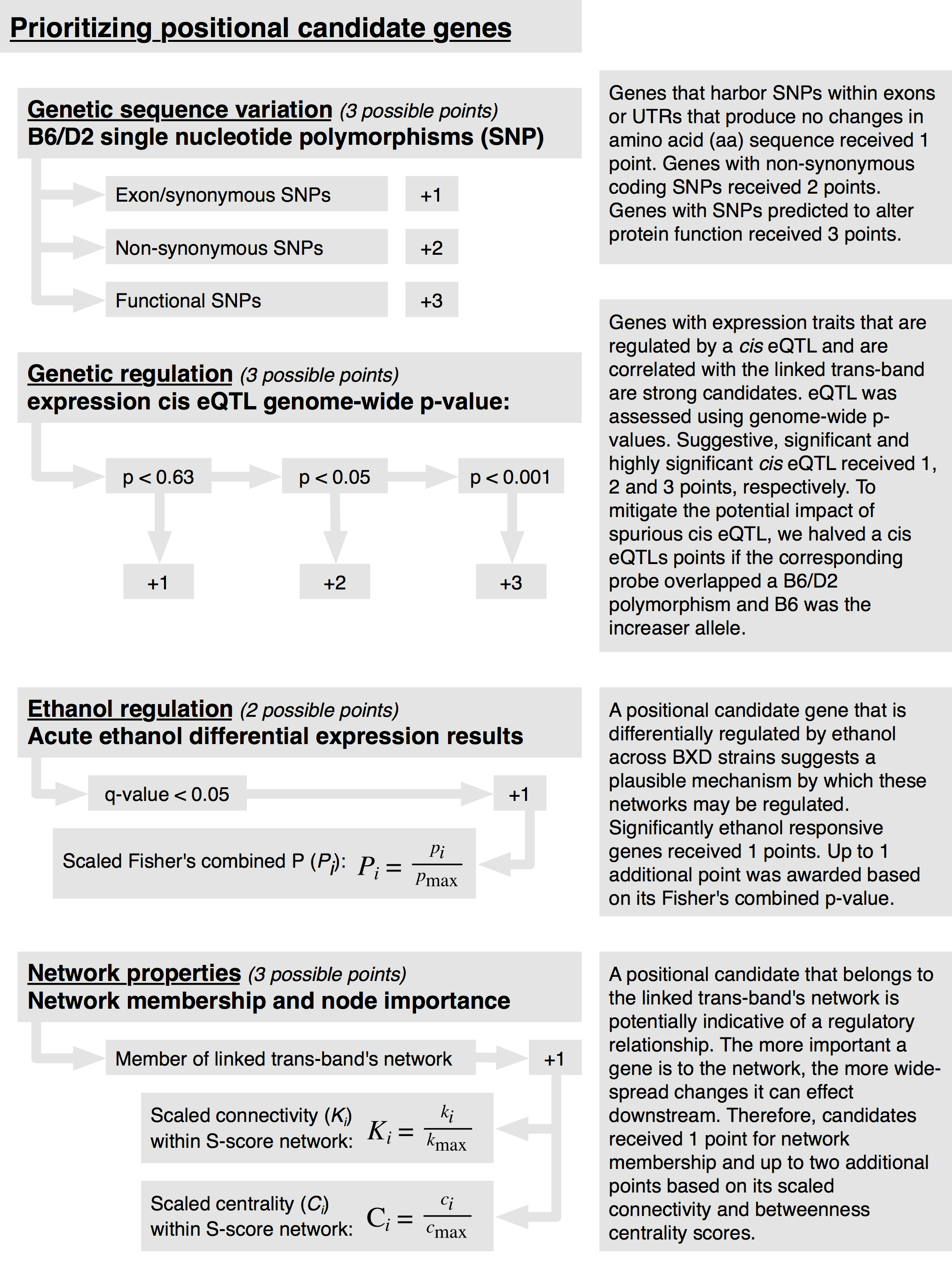

Supplement: Figure S1 — Outline of integrative strategy used to prioritize positional candidate genes underlying ErGeN trans-bands. (TIFF) [file pone.0033575.s001.tiff]

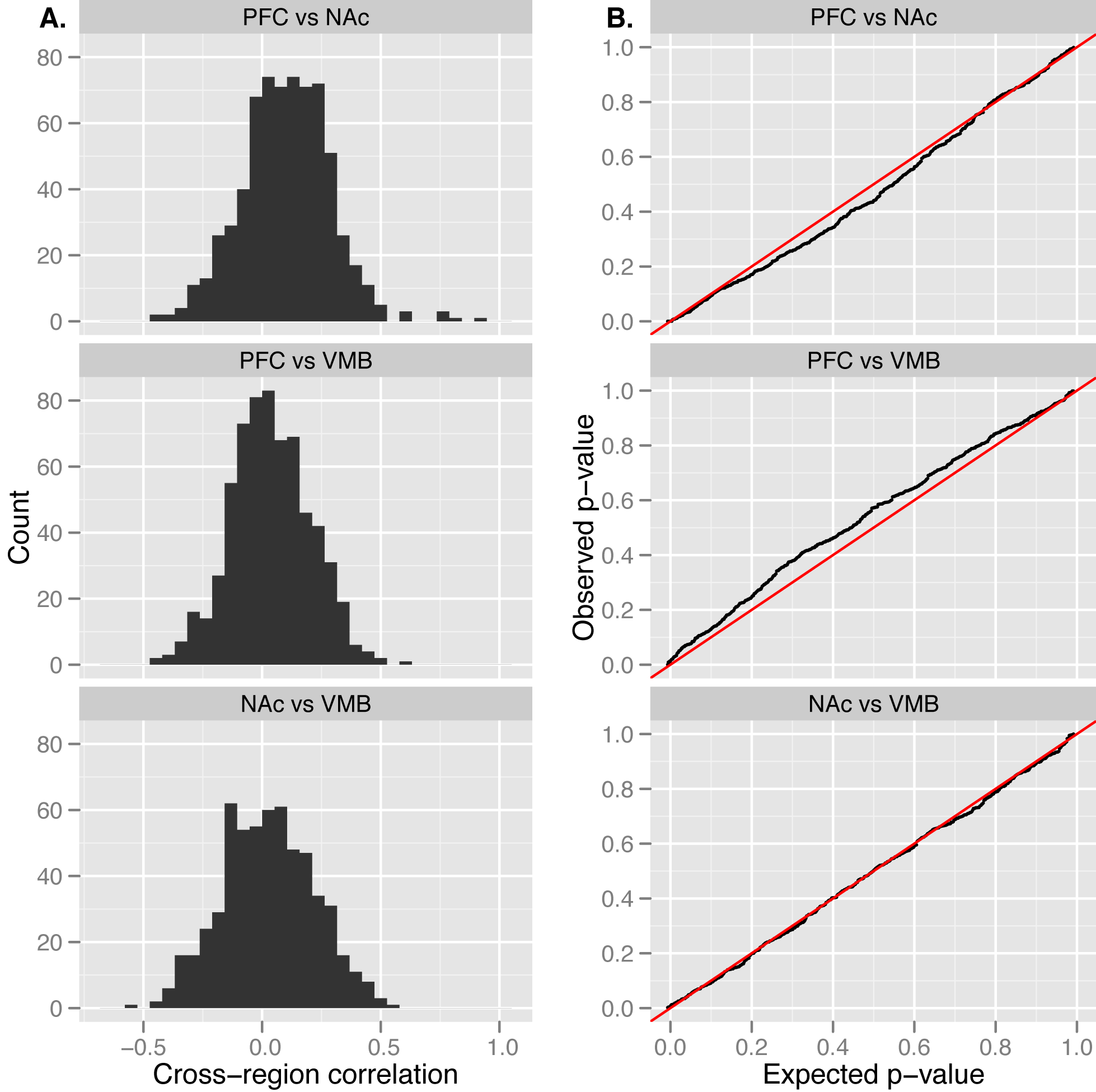

Supplement: Figure S2 — In order to determine the degree to which a gene's transcriptional response to ethanol is tissue-specific, we calculated cross-regional S-score correlations for each of the 399 probe-sets that were significantly ethanol responsive in the PFC, NAc and VMB. (A) The distribution of Pearson correlation coefficients and (B) corresponding p-values, indicate there is effectively no coordinated response to ethanol across regions. (TIF) [file pone.0033575.s002.tiff]

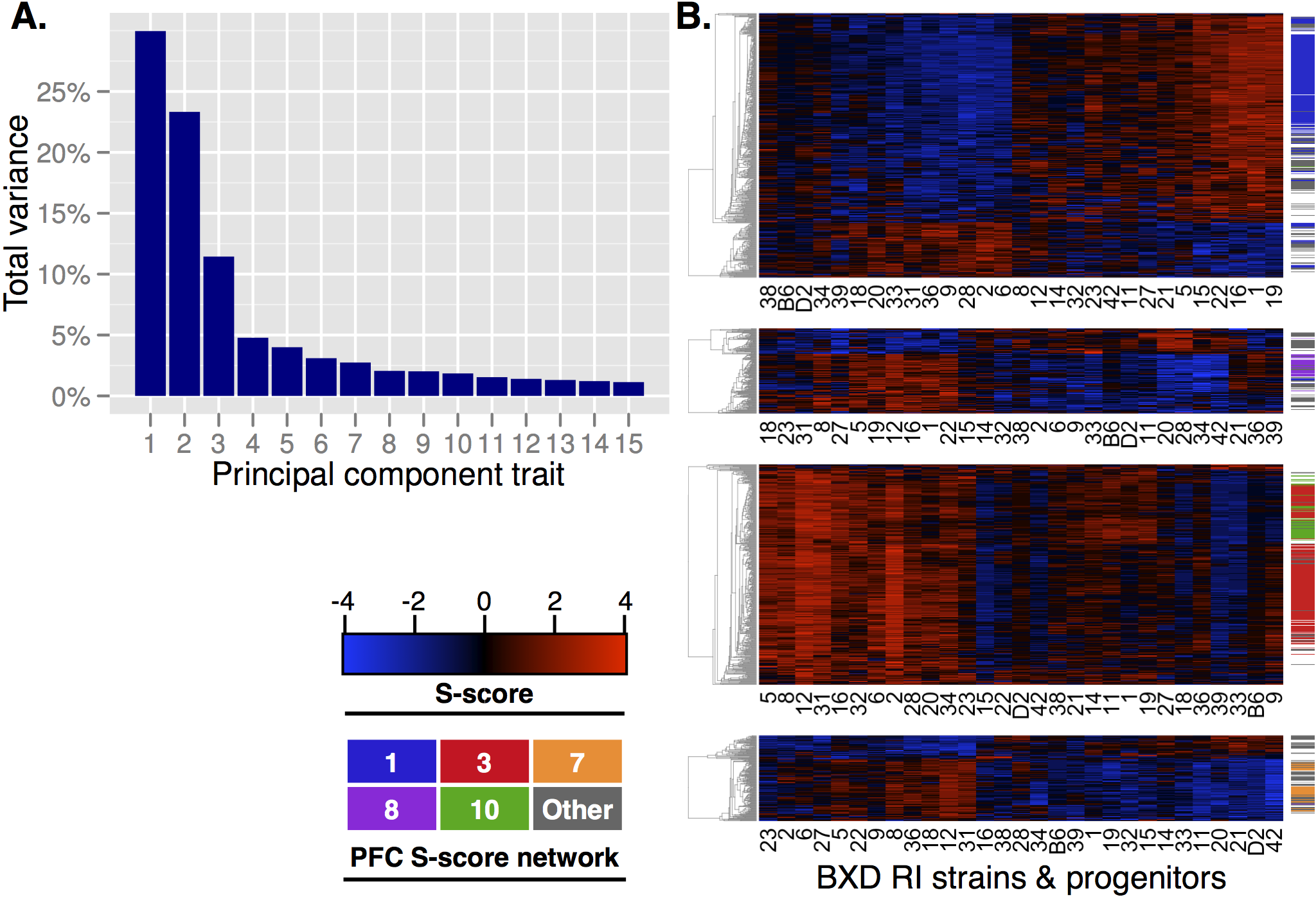

Supplement: Figure S3 — Traditional non-parametric partitioning and clustering of all significantly ethanol-responsive genes in the PFC. The number of modules was determined by principal component analysis, which revealed the first 4 components explained ∼70% of the variation in these genes S-scores (A). Genes were assigned to modules by partitioning around medoids, which were then independently hierarchically clustered based on average linkage of Pearson correlations. These results are visualized in the heatmap (B). Warmer colors represent positive S-scores (up-regulated by ethanol) whereas cooler colors indicate negative S-scores (down-regulated by ethanol). The adjacent column of colors indicates to which PFC S-score network the corresponding gene was assigned. (TIFF) [file pone.0033575.s003.tiff]

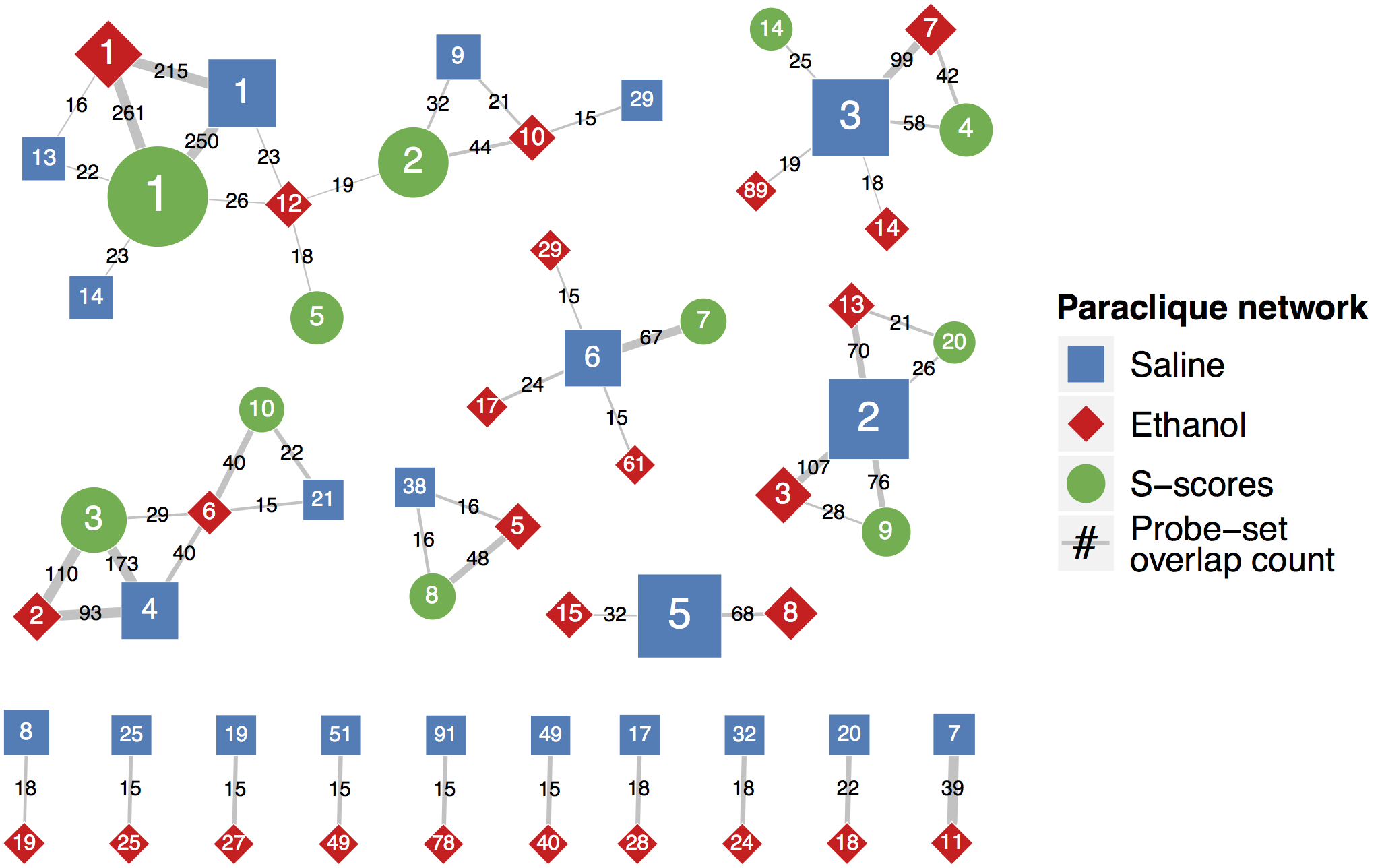

Supplement: Figure S4 — Substantial overlap existed between the gene constituents of paraclique networks formed with the saline (blue squares) and ethanol (red diamonds) RMA expression datasets, and the saline vs ethanol S-score (green circles) expression data. The statistical significance of overlap between networks was determined by Fisher's exact test, which identified 161 inter-dataset networks with more genes in common (edge numbers) than would be expected by chance, based on a Bonferroni-corrected p-value<0.05 (Table S4). The figure depicts a subset of the overlapping inter-dataset network relationships that share at least 15 genes in common. Each shape represents the co-expression network specified by its label. Node color and shape indicate the expression dataset used to form the network, while node size is proportional to the number of genes comprising the network. Edge thickness represents the statistical significance of the overlap. (TIF) [file pone.0033575.s004.tif]

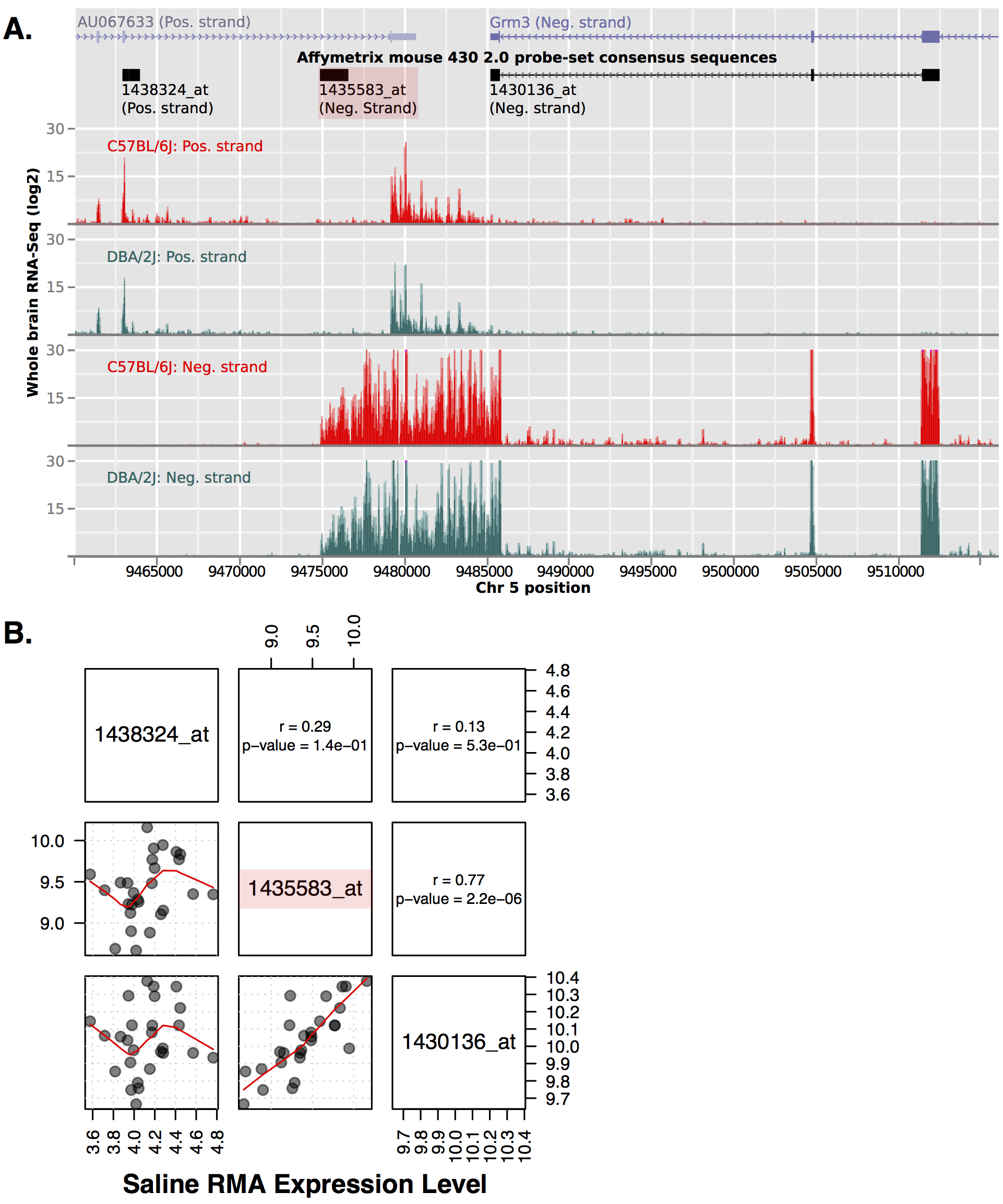

Supplement: Figure S5 — Whole brain RNA-Seq expression data across the Chr 5 region that encompasses AU067633 and Grm3 (A), adapted from GeneNetwork mirror of the UCSC Genome Browser (ucscbrowser.genenetwork.org). Although probe-set 1435583_at (red) putatively maps to an AU067633 intron, it appears to actually target Grm3's 3′ UTR, which is highly expressed from the negative strand across the same stretch of DNA. Probe-set 1435583_at's basal RMA expression levels were significantly correlated with the distal Grm3 probe-set, 1430136_at, while showing no relationship to the proximal AU067633 probe-set, 14338324_at (B). (TIFF) [file pone.0033575.s005.tiff]

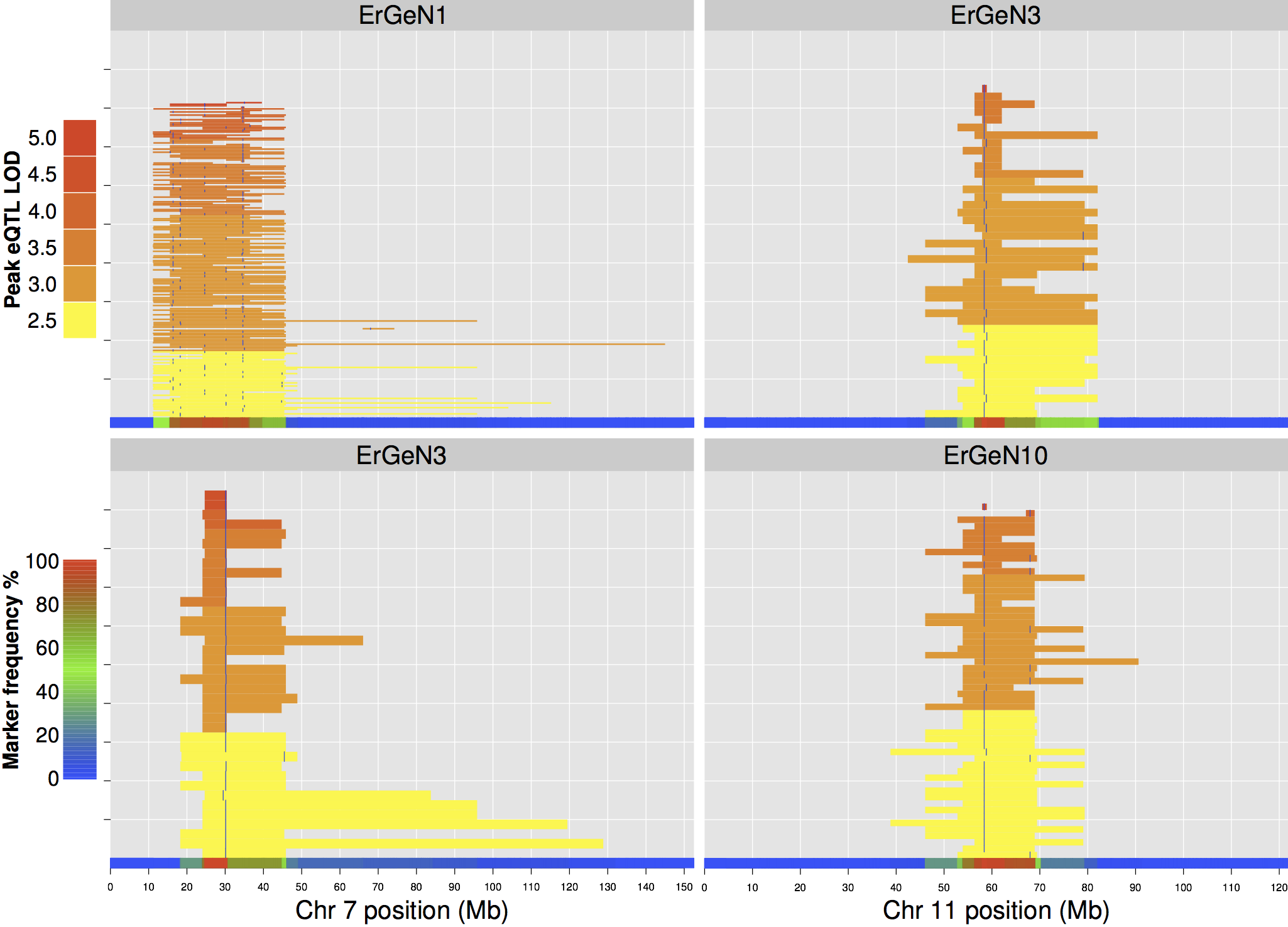

Supplement: Figure S6 — Support intervals for the major eQTL hotspot on Chr 7 for ErGeN1 (A) and ErGeN3 (B), and the eQTL hotspot on Chr 11 for ErGeN3 (C) and ErGeN10 (D). Each horizontal line represents an individual probe-set's 1.5 LOD drop support interval, ordered and colored based on peak LOD score. Blue ticks indicate peak eQTL locations. The heatmap along the x-axis represents the percentage of probe-set support intervals that encompass the underlying markers. (TIFF) [file pone.0033575.s006.tiff]

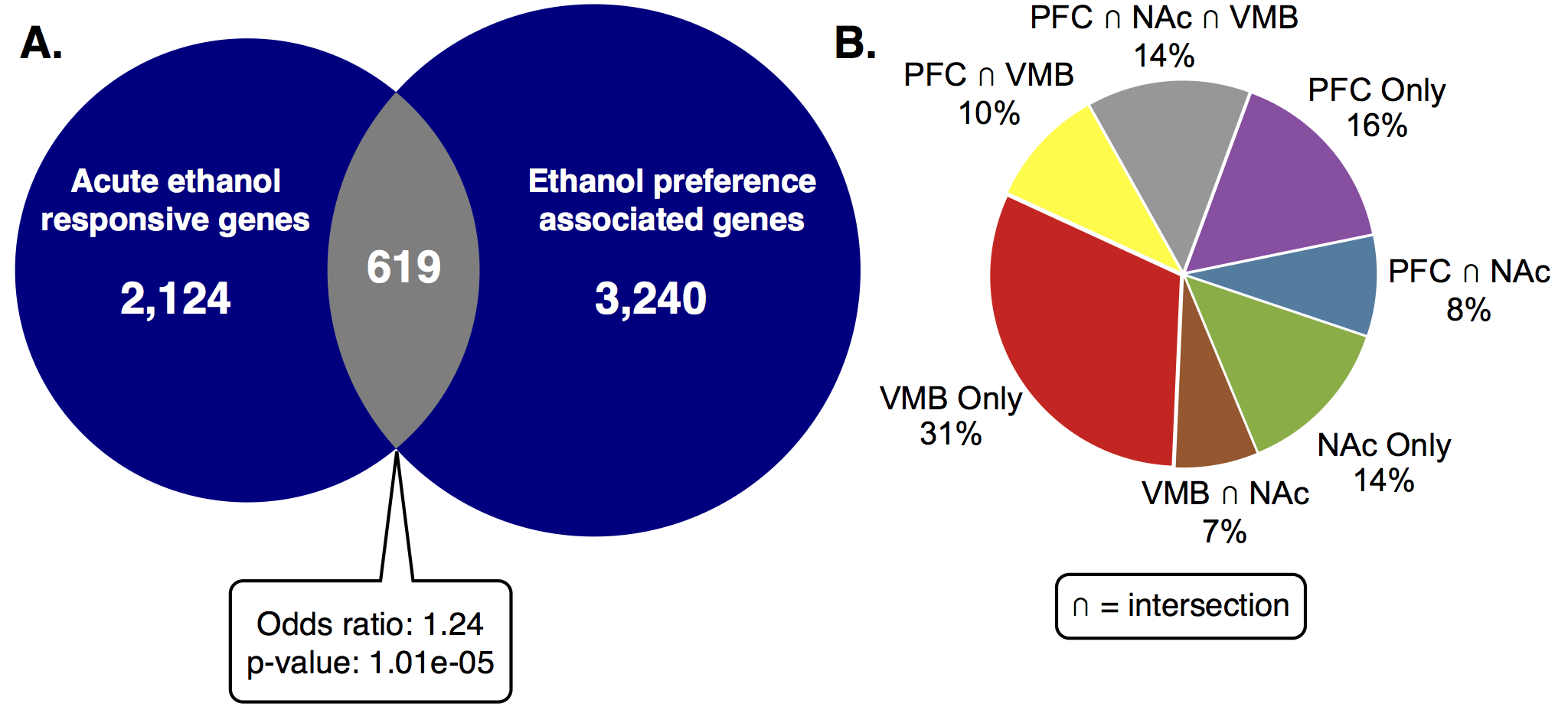

Supplement: Figure S7 — (A) Overlap between 2,743 genes that exhibited a significant response to acute ethanol across the BXD family in PFC, NAc or VMB and 3,859 genes identified as differentially expressed between several high and low ethanol preferring strains in a meta-analysis of whole brain tissue [70]. Overlap significance was measured using a one-tailed Fisher's exact test. (B) Distribution of the intersecting acute-ethanol/ethanol-preference genes among profiled regions of mesocorticolimbic CNS reward circuit. (TIFF) [file pone.0033575.s007.tiff]
